# Sn(II)/PN@AC catalysts: synthesis, physical-chemical characterization, and applications

**DOI:** 10.3906/kim-2103-9

**Published:** 2021-10-19

**Authors:** Yibo WU, Fuxiang LI, Qinbin LI, Yongjun HAN, Li WANG, Wei MA, Fu XV

**Affiliations:** 1 College of Chemistry and Environmental Engineering, Pingding Shan University, Pingding Shan China; 2 College of Chemistry and Chemical Engineering, Taiyuan University of Technology, Taiyuan China

**Keywords:** Tin-based catalysts, nitrogen elements, phosphorus elements, acetylene hydrochlorination

## Abstract

In this study, the novel tin-based catalysts (Sn(II)/PN@AC) were prepared using the phosphorus and nitrogen dual-modified activated carbon as support and SnCl_2_ as active compounds, as well as then evaluated in acetylene hydrochlorination. Under the reaction temperature of 180 °C and an acetylene gas hourly space velocity (GHSV-C_2_H_2_) of 30 h^–1^, the 15%Sn(II)/PN@AC-550 showed the initial acetylene conversion of 100% and vinyl chloride selectivity over 98.5%. Additionally, the deactivation rate of 15%Sn(II)/PN@AC-550 reached 0.47% h^–1^, which was lower than that of 15%Sn(II)/AC-550 (1.02% h^–1^), suggesting that PN@AC-550 as novel support can retarded the deactivation of Sn(II)/AC-550 catalysts during acetylene hydrochlorination. Based on the catalytic tests and characterization results (XRD, Raman, BET surface area, TEM, C_2_H_2_-TPD, H_2_-TPR, XPS, FT-IR, TGA, and ICP), it demonstrated that PN@AC-550 as support could effectively improve the dispersion of tin species, retard the formation of coke deposition, lessen the oxidation of SnCl_2_ during the preparation process, as well as relatively inhibit the leach of tin species during the reaction. By combing the FTIR results and Rideal–Eley mechanism, we proposed that that HSnCl_3_ was transition state of SnCl_2_ in catalysis acetylene hydrochlorination and then adsorbed the acetylene to produce the vinyl chloride.

## 1. Introduction

Tin-based catalysts for diverse application includes electrochemical CO_2_ reduction [1] and acetylene hydrochlorination (reaction equation: H_2_C=CH_2_+HCl=H_2_C=CHCl) [2–4], the latter of which is a significant technology to manufacture vinyl chloride. Despite the initial activity of tin-based catalysts is close to traditional mercury-based acetylene hydrochlorination catalysts, there is still an ongoing study in prolonging the lifetime. The tin-based acetylene hydrochlorination catalysts can be classified into three categories: bimetallic or multimetallic tin-based catalysts [2–8], organotin-based catalysts [9–11], and tin complex-based catalysts [4,12]. Given the easily sublimation of SnCl_4_ under industrially relevant process conditions [2], the efficient durability of SnCl_4_/AC in acetylene hydrochlorination can be prolonged by the incorporation of BiCl_3_ and CoCl_2 _[8]. Moreover, the Kocheshkov redistribution reaction of SnCl_4_ and Ph_3_ClSn can further improve the catalytic behavior of SnCl_4_/AC [10]. Later on, Guo et al. founded that the synergistic effect of SnCl_2_, ZnCl_2_, and Tb_4_O_7_ can strengthen stability of SnCl_2_/AC in acetylene hydrochlorination [5]. In the previous work, we founded that SnCl_2_/AC is easily oxidized during preparation process, but Li-Sn(IV) in LiSnCl_n_/AC is structurally stable, enhancing the stability of SnCl_2_/AC in acetylene hydrochlorination [3]. Moreover, based on the Rideal–Eley mechanism and the previous study and mentioned above results, we can infer that HSnCl_3_ is a transition state of tin active sites and then adsorbed the acetylene to produce the vinyl chloride [3]. Additionally, the lifetime of SnCl_2_/AC catalysts in acetylene hydrochlorination can be also elevated via the coordination interaction of organic linkage and Sn sites [4,11]. 

Owing to the dispersion of Cu species on catalysts surface keeps the direct relation with P doping, Cu-P/AC catalysts displays longer lifetime as compare to CuCl_2_/AC in acetylene hydrochlorination [13–15]. Also, nitrogen-doped in Cu-based catalysts for acetylene hydrochlorination can improve the acetylene adsorption and prolong the lifetime [16]. Combing the above results, it is demonstrated that dopants of nitrogen or phosphorus elements into Cu-based catalysts for acetylene hydrochlorination can strengthen the stability [13–18].

However, until now few studies researched the effect of nitrogen and phosphate elements on the catalytic performance of SnCl_2_/AC catalysts for acetylene hydrochlorination. In this work, Sn(II)/PN@AC was prepared using the low-cost phosphoric acid, melamine, and SnCl_2_ as phosphorus, nitrogen, and tin source, respectively. 

## 2. Materials and methods

### 2.1. Chemicals 

Coal-based carbon carrier (AC, 40-60 meshes) was bought from Shanxi Xinhua Chemical Company. Melamine (C_2_H_4_N_4_, ≥99.5%) and methyl orange was obtained from Tianjin Guangfu Technology Development Co., Ltd. Additionally, phosphoric acid (H_3_PO_4_ ≥98.0%) wea purchased from Sinopharm Chemical Reagent Co., Ltd. SnCl_2_·2H_2_O (≥98.0%) was bought from Energy Chemical Co., Ltd. Moreover, Na_2_CO_3_ (≥99.8%) was obtained from Tianjin Kermel Technology Development Co., Ltd. All materials were used without further purification.

### 2.2. Catalyst preparation 

H_3_PO_4_ (0.54 g), C_2_H_4_N_4 _(0.46 g), and AC (9.0 g) were firstly premixed in distilled water at 55 °C for 90 min, after which the sample was dried at 80 °C overnight. The obtained samples were calcinated at 550 °C for 4 h and then denoted as PN@AC. N@AC was prepared by the above-mentioned procedure.

Catalysts were prepared by the following processes. Firstly, SnCl_2_·2H_2_O (0.47 g) was completely dissolved in ethanol. Subsequently, SnCl_2_ solution was gradually added to carbon supports, followed by air drying at 80 °C for 12 h. The above-mentioned described procedures were repeated to prepare 10%Sn(II)/PN@AC, 10%Sn(II)/N@AC, and 10%Sn(II)/AC, respectively.

### 2.3. Catalytic performance

The catalytic performance was tested in a fixed-bed quartz reactor (i.d.=10 mm). To remove water vapor, the reaction system was washed by hydrogen chloride for 30 min before the initial reaction. Then the gas mixture of HCl and C_2_H_2_ (V_C2H2_/V_HCl _= 1.0:1.1) was introduced into reactor containing 4.0 mL of catalysts with C_2_H_2_-GHSV = 30 h^–1^ or 60 h^–1^ at 180 °C [19]. The final products contained the unreacted hydrogen chloride, which was adsorbed by the medical soda-lime. Having experienced adsorption, the cleaned gas mixture was analyzed online by GC900 (GDX-301 column).

### 2.4. Catalyst characterization 

The BET surface area and pore textural properties data of the catalysts was acquired by a nitrogen adsorption method using Quantachrome Nova2000e instruments. The X-ray diffraction (XRD) patterns of the catalysts was collected from a Shimadzu XRD-6000 instrument using Cu Ka radiation over the range of 10–80 °. Raman spectra of sample were performed on a Renishaw (514 nm laser source). Transmission electron microscopy (TEM) images were obtained using a JEM-2100F instrument with 300 keV acceleration voltages. X-ray photo-electron spectroscopy (XPS) analysis was conducted on an Esca Lab 250Xi spectrometer. Thermogravimetric analysis (TGA) experiments (NETZSCH STA 449F3 Jupiter instrument) carried out to study the coke deposition of catalysts. Inductively coupled plasma optical emission spectrometer (Agilent 720 ICP-OES) was used to determine the absolute content of tin elements in samples. 

The Fourier transform infrared spectra (FT-IR) of the samples was measured by Biorad Excalibur FTS 3000). Acetylene temperature-programmed desorption (C_2_H_2_-TPD) and hydrogen temperature-programmed reduction experiments (H_2_-TPR) were performed on FINESORB-3010 instruments, respectively [3]. Adsorption capacity of catalysts for HCl were calculated by the titration method [4]. 

## 3. Results and discussion 

### 3.1. Characterization of supports

**Figure 1:**
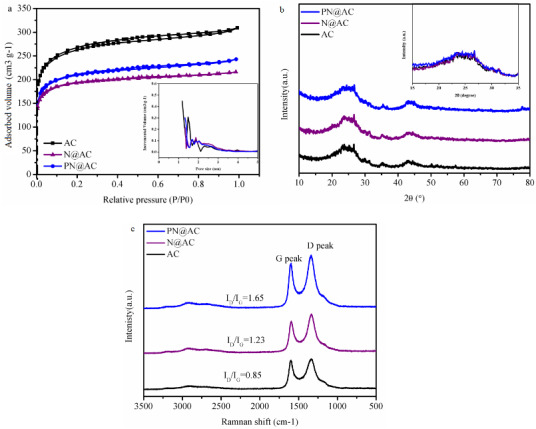
(a) N2 adsorption-desorption isotherms of samples; (b) X-ray diffraction patterns of samples; (c) Raman spectra of samples.

The structure of different supports was firstly studied, including the BET surface area and the pore volume. The value of AC (983.0 m^2^·g^–1^, 0.48 m^3^·g^–1^) was higher than that of N@AC (786.3 m^2^·g^–1^, 0.38 m^3^·g^–1^) and PN@AC (725.1 m^2^·g^–1^, 0.33 m^3^·g^–1^). It is demonstrated that the nonmetal additives are filled into the partial pores of AC (Figure 1a and Table 1). Figure 1b shows that the two diffraction peaks of AC were not affected by the introduction of nonmetal additives [20], inferring the well dispersion of promoter on AC surface [21]. As shown in Figure 1c, the I_D_/I_G_ value gradually declines in the order of PN@AC(1.63)> N@AC(1.23)> AC (0.85), suggesting that PN-doping does lead to the more abundant defect of PN@AC and consequently improve the catalytic behavior of AC in acetylene hydrochlorination [22–24].

**Table 1:** Textural properties of different catalysts.

Samples	SBET(cm2 g–1)	Smicro(cm2 g–1)	Smeso(cm2 g–1)	Total volume(cm3 g–1)	D(nm)
AC	983.0	853.9	129.1	0.48	1.9
N@AC	786.3	701.2	85.1	0.38	1.9
PN@AC	725.1	667.4	57.7	0.33	1.8
5%Sn(II)/PN@AC	547.8	485.6	62.2	0.26	1.9
10%Sn(II)/PN@AC	459.5	395.5	64.0	0.23	2.0
15%Sn(II)/PN@AC	303.3	203.2	67.9	0.20	2.1
20%Sn(II)/PN@AC	155.5	104.9	50.6	0.10	2.4

### 3.2. Characterization of Sn-based catalysts

**Figure 2:**
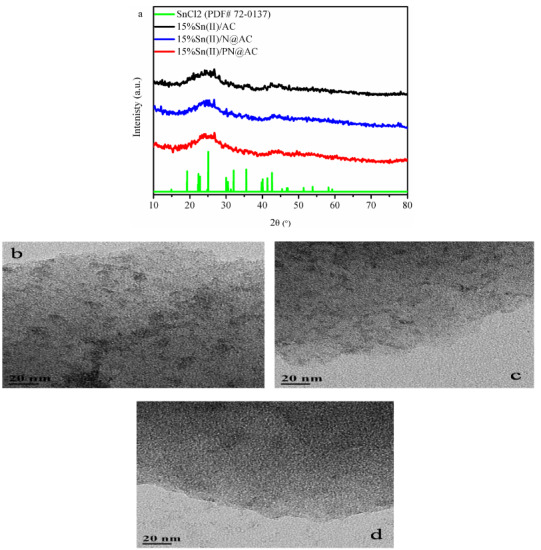
(a) X-ray diffraction patterns of catalysts; TEM images of (b) 15%Sn(II)/AC, (c) 15%Sn(II)//N@AC, and (d) 15%Sn(II)/PN@AC.

As evident from Table 1 and Table 2, the load of SnCl_2_ can reduce the specific surface area and the pore volume of supports. Furthermore, besides the characteristic peaks of carbon, the discernible peaks of SnCl_2_ (PDF#72-0137) is not observed in tin-based catalysts (Figure 2a), inferring the dispersion of tin compounds on support surface [20,21]. As shown in Figures 2b and 2c, numerous black particles are dispersed on the support surface. Meanwhile, Figure 2d illustrates that no large particles are reunited on the PN@AC, indicating that Sn species are stably and homogeneously dispersed on the PN@AC. 

**Table 2:** Textural and structure properties of tin-based catalysts.

Sample	SBET(m2·g–1)		ΔSBET(m2·g–1)	Vtotal(m3·g–1)	
	Fresh	Used		Fresh	Used
15%Sn(II)/AC	390.9	91.2	299.7	0.15	0.07
15%Sn(II)/N@AC	362.2	121.1	241.1	0.18	0.09
15%Sn(II)/PN@AC	303.3	164.7	138.6	0.20	0.14

### 3.3. Catalytic performance

**Figure 3:**
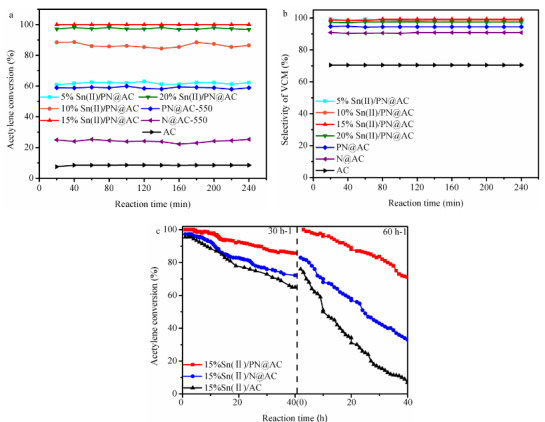
Acetylene conversion (a) and VCM selectivity (b) of catalysts (reaction conditions: T = 180 oC, C2H2-GHSV=30 h–1 and VHCl/VC2H2 =1.1/1.0) (c) The stability of 15%Sn(II)/PN@AC, 15%Sn(II)/N@AC and 15%Sn(II)/AC (Reaction conditions: T = 180 oC, C2H2-GHSV=30 h–1 or 60 h–1 and VHCl/VC2H2 =1.1/1.0)

The catalytic performances of different catalysts were tested in acetylene hydrochlorination, and the results are shown in Figures 3a-c. The acetylene conversion on the Sn(II)/PN@AC increased as the SnCl_2_ content (5 wt%–15 wt%). When the SnCl_2 _content is 15 wt%, Sn(II)/PN@AC achieves an initial acetylene conversion of 100%, with vinyl chloride selectivity of 98.5% (Figures 3a and 3b). It is noted that the acetylene conversion on 20%Sn(II)/PN@AC does not increase but decrease to 96.8%. However, Sn(II)/AC only behaved about 95.5% acetylene conversion (Figure 3c). At same time, the VCM selectivity of Sn(II)/PN@AC is over 98.0%. The results indicated that Sn(II)/PN@AC exhibited highly selectivity and catalytic activity in acetylene hydrochlorination. After 40 h reaction (Figure 3c), the acetylene conversion of 15%Sn(II)/AC is 62.1%, while the acetylene conversion of 15%Sn(II)/N@AC decreases from 97.3% to 72.2%, indicating that synergy between Sn species and N is one reason for the longer lifetime. Notably, 15%Sn(II)/PN@AC has an unusual catalytic stability in acetylene hydrochlorination reaction compared to 15%Sn(II)/AC catalysts with the same SnCl_2 _loading, which indicates that the dual-elements (N and P) additives can further strengthen the stability of 15%Sn(II)/AC (Figure 3c). For 15%Sn(II)/N@AC and 15%Sn(II)/AC, the acetylene conversion under C_2_H_2_-GHSV of 60 h^–1 ^was 82.7% and 78.5%, respectively, while for 15%Sn(II)/PN@AC, the acetylene conversion under the same reaction condition (60 h^–1^) was 99.7% (Figure 3c). Overall, 15%Sn(II)/PN@AC behaves the acetylene conversion of 99.0%, which meets the industrial production need (C_2_H_2_-GHSV=30 h^–1 ^-50 h^–^^1 ^and T = 130 ^o^C -180 ^o^C ) [25].

### 3.4. Synergistic effect between Sn and supports

**Figure 4:**
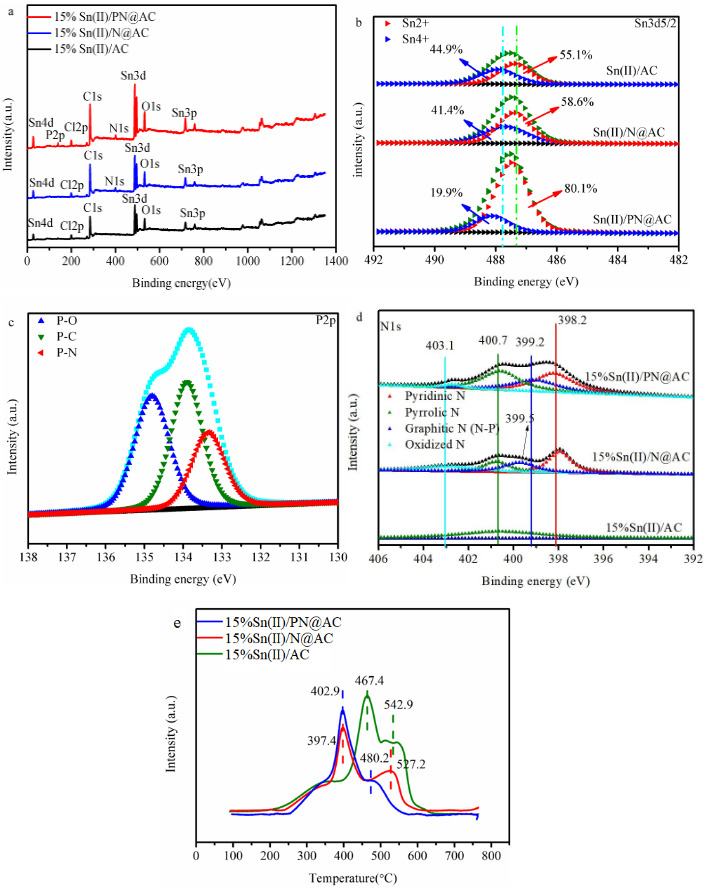
(a) XPS pattern of catalysts; (b) XPS-Sn3d5/2 pattern of catalysts; (c) XPS-P2p pattern of 15%Sn(II)/PN@AC; (d) XPS-N1s pattern of catalysts; (e) H2-TPR spectra of samples.

The status of tin, nitrogen and phosphorus species in catalysts was characterized by XPS analysis (Figure 4a and Table 3). As Figure 4b illustrates, Sn-based catalysts behaved the two types of Sn species including Sn^2+ ^(~486.7 eV) and Sn^4+^(~487.7 eV) [26–28]. This result is consistent with H_2_-TPR results (Table 4). As shown in Figure 4e and Table 4, the H_2_ reduction peaks of Sn^2+^ and Sn^4+ ^in catalysts (15%Sn(II)/N@AC and 15%Sn(II)/PN@AC) was lower than the standard consumption peaks (467.4 and 542.9 °C) [26–28], inferring that the nonmetal element additives can retard the oxidation of Sn^2+ ^during the synthesis process and lessen the leach of Sn species during the reaction. The determination of Sn^2+^ and Sn^4+ ^values in Tables (Table 4 and Table 5) was calculated on the normalization of peak areas. Figure 4c and Figure 4d confirm the existence of N-P and Pyridinic N, which can promote the reactivity of catalysts significantly [25,29,30]. The Pyridinic N content of 15%Sn(II)/N@AC was calculated to be 0.9 wt%, which is close to the 15%Sn(II)/PN@AC (0.88 wt%). It is revealed that phosphorus atoms bonded with nitrogen in the pyridine structure (ortho-position NP) is the main factor on the performance of 15%Sn(II)/AC according to the present work (Figure 1a, Figure 4d, and Table 6) and a recently published paper [25]. 

**Table 3:** Surface chemical element content of different catalysts by XPS.

Sample	Composition (wt%)					
	Sn	C	O	P	N	Cl
15%Sn(II)/AC	8.55	75.68	16.74	0.07	0.44	5.23
15%Sn(II)/N@AC	8.67	74.27	15.88	0.05	2.52	4.23
15%Sn(II)/PN@AC	8.90	72.66	16.42	0.91	2.43	4.64

**Table 4:** The relative peak area (TPR) of Sn2+ and Sn4+ in different tin-based catalysts.

Samples	Peak area (%)	
	Sn2+	Sn4+
15%Sn(II)/AC	53.2(467.4 °C)	45.8(542.9 °C)
15%Sn(II)/N@AC	69.7(397.4 °C)	30.3(527.2 °C)
15%Sn(II)/PN@AC	86.2(402.9 °C)	13.8 (480.2 °C)

**Table 5:** The content of Sn2+ and Sn4+ in different tin-based catalysts.

Sample	Composition (wt %)		
	Total Sn	Sn4+	Sn2+
15%Sn(II)/AC	4.55	2.04	2.51
15%Sn(II)/N@AC	4.67	1.93	2.74
15%Sn(II)/PN@AC	4.90	0.98	3.92

**Table 6:** Chemical composition of the N1s in different tin-based catalysts (XPS).

Samples	Composition (wt %)				
	TotalN	PyridinicN	GraphiticN	PyrrolicN	OxidizedN
15%Sn(II)/AC	0.44	--	0.44	--	--
15%Sn(II)/N@AC	2.52	0.90	0.81	0.64	0.17
15%Sn(II)/PN@AC	2.43	0.88	0.84	0.62	0.09

As shown in Figure 4c, the electron binding energies at 135.0 eV, 133.5 eV, and 132.6 eV can be assigned to P-O, P-N, and P-C [23,24,31]. Particularly, the addition of SnCl_2_ into catalysts shift the P-N binding energy from 133.5 eV to 133.2 eV. Moreover, a positive shift (0.3 eV) can be observed in the binding energy of Sn^2+^ for 15%Sn(II)/PN@AC versus 15%Sn(II)/AC. Therefore, it is attributing this shift to the synergistic effect between ortho-position NP and Sn species [32,33].

### 3.5 Synergistic effect between Sn and reactants

**Figure 5:**
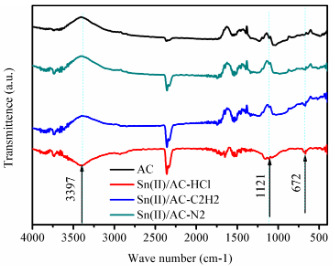
FT-IR spectra of samples.

To study the effect of various adsorption reactants on the structural conformation of SnCl_2_, we used FT-IR techniques to analyze three samples including Sn(II)/AC-C_2_H_2_, Sn(II)/AC-HCl, and Sn(II)/AC-N_2_ (Figure 5), which represent that SnCl_2_/AC was respectively pretreated at 200 °C for 1 h under acetylene, hydrogen chloride, and nitrogen atmosphere, respectively. Two characteristic adsorption bands at ~1121 cm^–1^ and ~3397 cm^–1^ are observed in Sn(II)/AC-HCl, suggesting that the gaseous HCl reacted firstly with Sn species. Figure 4a shows that Sn(II)/AC catalysts during acetylene hydrochlorination contains SnCl_4 _and SnCl_2_, the latter of which is the main formation of Sn species. Based on the analysis of our previous study [3], owing to SnCl_4_ and HCl are electron-receptor, the adsorption of HCl on the SnCl_2_ sites forms the HSnCl_3 _[34–36]. The results of FT-IR spectra are no obvious difference between Sn(II)/AC-N_2 _and Sn(II)/AC-C_2_H_2_, implying that SnCl_2_ cannot make bond with C_2_H_2_. Combining the Eley–Rideal mechanism and previous work [34–38], it is indicated that HSnCl_3_ is as transition state of SnCl_2_ in the catalysis of acetylene hydrochlorination.

**Figure 6:**
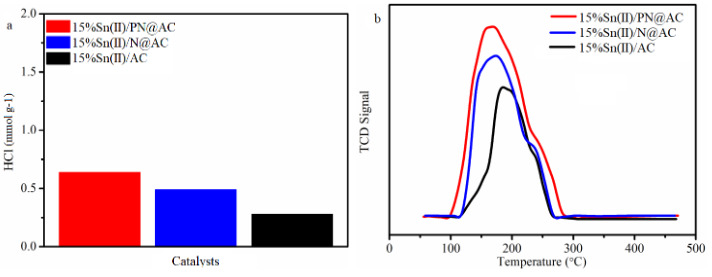
(a) HCl adsorption and (b) C2H2-TPD profile of catalysts.

HCl adsorption experiments was applied to study the HCl adsorption capacity of catalysts, and the results are displayed in Figure 6a. The adsorption capacity of HCl on 15%Sn(II)/PN@AC, 15%Sn(II)/N@AC, and 15%Sn(II)/AC are 0.64 mmol·g^–1^, 0.49 mmol·g^–1^, and 0.28 mmol·g^–1^, respectively. The published papers finds that HCl adsorbs on Pyridinic N firstly [30], but the Pyridinic N content in 15%Sn(II)/PN@AC is close to 15%Sn(II)/N@AC (Table 6). It is implying that the synergy effect between Sn and NP may be responsible for the highly HCl adsorption capacity and thus improve the catalytic behaviors of tin-based catalysts in acetylene hydrochlorination, based on the above results.

As shown in Figure 6b, the adsorption area for C_2_H_2 _of catalysts decreases in the order 15%Sn(II)/PN@AC>15%Sn(II)/N@AC>15%Sn(II)/AC. When N and P-codoped 15%Sn(II)/AC can result in higher C_2_H_2 _adsorption capacity as compare to 15%Sn(II)/N@AC and 15%Sn(II)/AC. This result indicates that NP is responsible for the C_2_H_2_ adsorption capacity, which is well agree with the previous study [25].

### 3.6 Inactivation of tin-based catalysts

**Figure 7:**
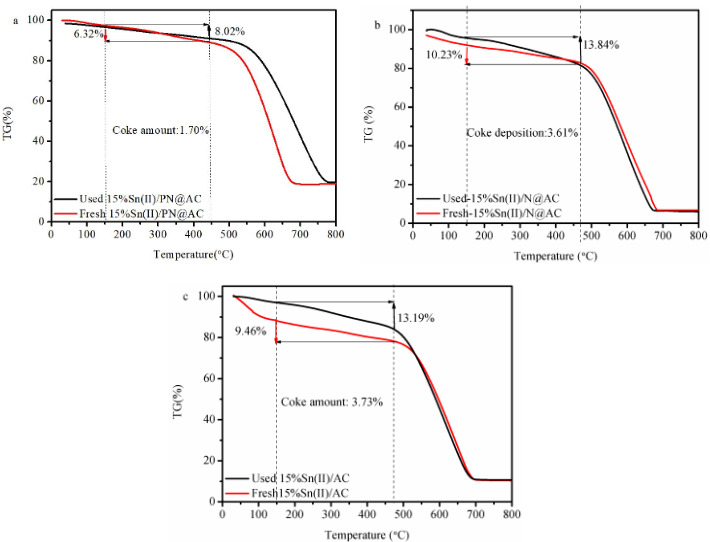
TG curves of catalysts: (a) 15%Sn(II)/PN@AC; (b) 15%Sn(II)/N@AC; (c) 15%Sn(II)/AC.

After 40 h reaction, the reduction of BET surface area in used tin-based catalysts is listed in Table 2, the loss of BET surface area in 15%Sn(II)/PN@AC, 15%Sn(II)/N@AC and 15%Sn(II)/AC reaches 138.6 m^2^·g^–1^, 241.1 m^2^·g^–1^, and 299.7 m^2^·g^–1^. Figure 7 shows the TGA curves of catalysts before and after reaction. Based on the same type fresh- and used-catalysts, the weight loss in the temperature range of 150–470 °C was mainly originated from the coke deposition on the catalysts surface in acetylene hydrochlorination [39,40], and the results is listed in Table 7. The amount of coke deposition for the 15%Sn(II)/PN@AC, 15%Sn(II)/N@AC, and 15%Sn(II)/AC catalysts are 1.70%, 3.61%, and 3.70% (Figures 7a and b). Thus, the synergistic effect of Sn species and NP in 15%Sn(II)/PN@AC can accelerate the anti-coking ability, which, consequently, prolonging the lifetime of the Sn-based catalysts. The 75.9%, 72.3%, and 65.4% of the initial Sn content is lost from 15%Sn(II)/AC, 15%Sn(II)/N@AC, and 15%Sn(II)/PN@AC, respectively, after 40 h reaction (Table 8), demonstrating that the leach of tin species is a deactivation reason of tin-based catalysts and manifesting that NP additives can retard the loss of tin compounds during acetylene hydrochlorination[41]. 

**Table 7:** Coke deposition of catalysts.

Samples	Coke deposition (%)
15%Sn(II)/AC	3.73
15%Sn(II)/N@AC	3.61
15%Sn(II)/PN@AC	1.70

**Table 8:** The content of Sn in fresh and used tin based catalysts determined by different method.

Samples	Nominala(wt%)	ICP (wt%)		Loss ratio of Sn
		Fresh	Used	(%)
15%Sn(II)/AC	8.5	8.3	2.0	75.9%
15%Sn(II)/N@AC	8.5	8.3	2.3	72.3%
15% Sn(II)/PN@AC	8.5	8.1	2.8	65.4%

aTheoretical calculation method: M (%) = (Msn species /Msn)/(Mcatalysts).Msn species: the weight of Sn species; Msn: 225 g/mol; Mcatalysts

## 4. Conclusion

Although carbon supported-HgCl_2_ as catalyst exhibits considerable catalytic performance, its toxicity and sublimation can be led to the serious environmental pollution. Additionally, the Minamata convention will be forbidden the utilization of mercury-based materials. But this study reported that Sn/PNAC as catalysts for acetylene hydrochlorination was prepared using nontoxic compounds. This work also finds that the stability of 15%Sn(II)/PN@AC catalysts correlate with N and P additives. After careful characterizations and additional catalytic tests, PN@AC supports can not only make the tin compounds dispersion well, but also strengthens the reactants adsorption of catalysts. According to the XPS, C_2_H_2_-TPD, H_2_-TPR, and HCl adsorption experiments results, it is found that the better catalytic behavior of Sn(II)/PN@AC is mainly attributed to the synergy between ortho-position NP and Sn species. Additionally, the reaction mechanism was proposed as follows, the adsorption of HCl on SnCl_2_ forms the transition state (HSnCl_3_) is the initial step and then react with C_2_H_2_ to produce the vinyl chloride. Such a finding provides the guidance to develop the tin-based catalysts for acetylene hydrochlorination. 
